# Identification and Characterization of a New Orthoreovirus from Patients with Acute Respiratory Infections

**DOI:** 10.1371/journal.pone.0003803

**Published:** 2008-11-25

**Authors:** Kaw Bing Chua, Kenny Voon, Gary Crameri, Hui Siu Tan, Juliana Rosli, Jennifer A. McEachern, Sivagami Suluraju, Meng Yu, Lin-Fa Wang

**Affiliations:** 1 National Public Health Laboratory, Sg. Buloh, Selangor, Malaysia; 2 International Medical University, Bukit Jalil, Kuala Lumpur, Malaysia; 3 CSIRO Livestock Industries, Australian Animal Health Laboratory and Australian Biosecurity Cooperative Research Center for Emerging Infectious Diseases, Geelong, Australia; 4 Klinik Kesihatan Kampar, Jalan Degong, Perak, Malaysia; Institut Pasteur, France

## Abstract

First discovered in the early 1950s, reoviruses (***r***espiratory ***e***nteric ***o***rphan viruses) were not associated with any known disease, and hence named orphan viruses. Recently, our group reported the isolation of the Melaka virus from a patient with acute respiratory disease and provided data suggesting that this new orthoreovirus is capable of human-to-human transmission and is probably of bat origin. Here we report yet another Melaka-like reovirus (named Kampar virus) isolated from the throat swab of a 54 year old male patient in Kampar, Perak, Malaysia who was suffering from high fever, acute respiratory disease and vomiting at the time of virus isolation. Serological studies indicated that Kampar virus was transmitted from the index case to at least one other individual and caused respiratory disease in the contact case. Sequence analysis of the four small class genome segments indicated that Kampar and Melaka viruses are closely related. This was confirmed by virus neutralization assay, showing an effective two-way cross neutralization, i.e., the serum against one virus was able to neutralize the other. Although the exact origin of Kampar virus is unknown, epidemiological tracing revealed that the house of the index case is surrounded by fruit trees frequently visited by fruit bats. There is a high probability that Kampar virus originated from bats and was transmitted to humans via bat droppings or contaminated fruits. The discovery of Kampar virus highlights the increasing trend of emergence of bat zoonotic viruses and the need to expand our understanding of bats as a source of many unknown viruses.

## Introduction

Reoviruses (***r***espiratory ***e***nteric ***o***rphan viruses), members of the family *Reoviridae*, are a large and diverse group of non-enveloped viruses with segmented dsRNA genomes, which are taxonomically classified into ten genera [1], [2]. Members of the genus *Orthoreovirus* contain 10 genome segments and have been isolated from a broad range of mammalian, avian and reptilian hosts. Although orthoreoviruses have been identified as the causative agents of diseases in animals, infections in humans are generally benign with very rare cases of mild upper respiratory tract illness or enteritis in infants or children [3]. Orthoreoviruses are divided into two subgroups, fusogenic and nonfusogenic, based on the ability of the virus to induce cell-cell fusion and syncytium formation [4].

A fusogenic orthoreovirus, the Melaka virus (MelV), was isolated from a human patient suffering acute upper respiratory disease [5]. MelV was shown to be capable of human-to-human transmission and has close sequence relatedness to two bat-borne orthoreoviruses, the Nelson Bay virus (NBV) isolated from fruit bats in Australia and the Pulau virus (PulV) isolated from fruit bats in Malaysia [6], [7]. Epidemiological tracing suggested that MelV originated from bats and was transmitted directly to the index case, followed by subsequent transmission to other members of the same family [5].

Bats have been shown to be the reservoir hosts of many recently emergent zoonotic viruses, including Hendra virus, Nipah virus, Menangle virus, and potentially SARS and Ebola viruses [8]–[13]. NBV was the first reovirus of bat origin, which was isolated in 1968 from the heart blood of a flying fox (*Pteropus poliocephalus*) in New South Wales, Australia [6], [14]. NBV was also the first mammalian reovirus to display fusogenic properties [6], a characteristic previously only known for avian reoviruses (ARVs). In 1999, during a search for Nipah virus in pteropid bats on Tioman Island, PulV was isolated from *Pteropus hypomelanus* [7], [15].

Here, we report the discovery and characterization of Kampar virus (KamV), the fourth member in the NBV species group and its isolation from a human patient with fever and acute respiratory illness. Although there is no direct evidence to suggest that KamV originated from bats, the close relationship of KamV with other members of the NBV group and preliminary epidemiological data suggest that KamV is most likely a bat-borne orthoreovirus.

## Results

### Clinical symptoms and case history of patients involved in this study

To mitigate the potential health as well as socio-economic impact of emerging diseases, in particular, the potential emergence of pandemic influenza, the Ministry of Health Malaysia undertook nation-wide influenza-like illness surveillance for early detection, identification and control of these emerging diseases. In August 2006, as part of the surveillance process, a patient with acute influenza-like illness was investigated in Kampar, a town situated in the north-western part of peninsular Malaysia, about 36 kilometres south of Ipoh, the capital city of the state Perak.

#### Case 1 (index case)

Subject 1 (S1), a 54-year old Chinese man, was well until 19 August 2006 when he developed sudden onset of high fever with chills and rigor. This was associated with cough, sore-throat and headache. There was no associated dyspnoea, tachypnoea, haemoptysis or chest pain on coughing. Besides the severe frontal throbbing headache, he had generalized body aches, myalgia and severe malaise. On the following day, he developed nausea, vomiting and diarrhoea. The vomitus consisted of food taken and was not bile-stained. The stool was described as watery without excessive mucous and it was non-malenic. The gastrointestinal symptom was associated with abdominal pain and loss of appetite. His illness was not relieved with self-medication of antipyretics. On review, there was no associated giddiness, blurring of vision, photophobia, skin bleeding or arthritis.

He sought medical treatment at the government health clinic in Kampar on 21 August 2006. At the outpatient clinic, he was noted to be febrile (an axillary temperature of 40.1 degree Celsius), ill-looking with a generalized body erythema that blanched on pressure and was more prominent over the face and upper trunk. He had mild conjunctivitis but there was no jaundice. His tonsils were enlarged and injected but there was no white exudate noted over the tonsils. He was not in any respiratory distress and his lungs were clear with good air entry on auscultation. Other systemic examination was essentially normal and there was no significant lymphadenopathy noted. A provisional diagnosis of influenza-like illness was made at which he was given a higher dose of anti-pyretic. His illness was noted to resolve on 23 August 2006 although he still appeared weak and lethargic.

Venous blood samples were taken from the patient for full blood count analysis, and the results are shown in Table 1. These results indicated that his white blood cell and platelet counts were within normal limits although there was a relative lymphopenia in the blood sample taken at first examination.

**Table 1:** Results of serial full blood count of the index patient with acute upper respiratory illness due to Kampar virus.

Blood Parameter	Date		
	August 21	August 22	August 23
Hemoglobin (g/dL)	13.6	14.5	14.6
Platelet count (/µL)	161 000	139 000	140 000
Total white blood cells (/µL)	5800	6500	5700
Neutrophil (%)	66.4	53.4	56.8
Lymphocyte (%)	18.1	35.4	36.1
Monocyte (%)	15.5	11.2	7.1

His throat swab was taken on August 21 for virus isolation as described below.

#### Case 2 (contact case)

Subject 2 (S2) is a 28 year old female Chinese medical officer who attended to the index patient in the government health clinic in Kampar on 21 August 2006. On 25 August, she developed nasal obstruction with mild runny nose. This was associated with a mild sore-throat and hoarseness of voice. There was no associated cough or breathing difficulty. Apart from the subjective feeling of mild lethargy and general unwellness, she did not experience fever, headache or myalgia. Her upper respiratory symptoms resolved within three days.

#### Case 3 (suspected contact case)

Subject 3 (S3), a 46 year old female, is the wife of the index patient. She regularly stays and takes care of the family home. Due to the nature of their work in other parts of the country, her husband and their 17-year old son only come back intermittently to stay in the same house. She was unable to re-call having a similar illness as her husband previously, although she did remember experiencing incidents of mild headache and fever in late August to early September, which resolved with self-medication. Since she did not seek medical help, there was no record of medical examination at the time of suspected infection.

### Isolation of a new virus from a patient with acute respiratory infection

**Figure 1:**
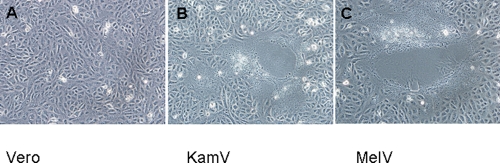
Cytopathic effect observed in Vero cells infected with KamV and MelV. A. Mock infected; B. KamV infected; C. MelV infected.

**Figure 2:**
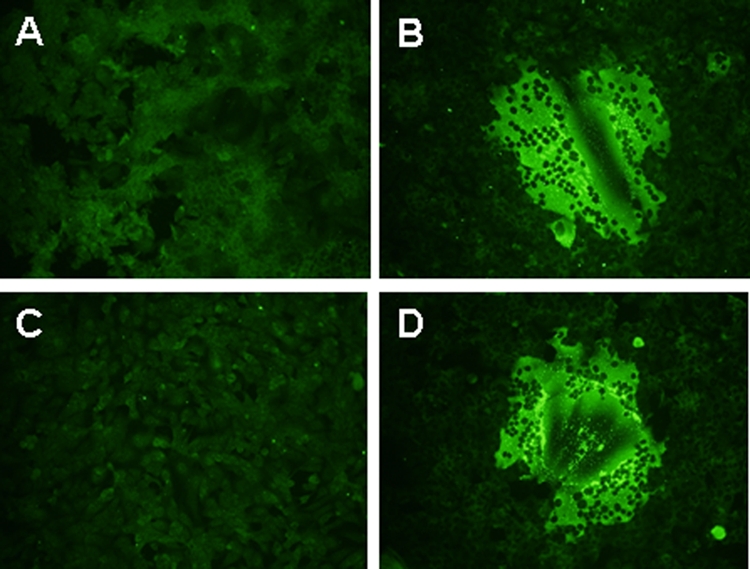
Analysis of cross-reactivity between KamV and MelV by immunofluorescent antibody test. Panels A and B are mock and KamV infected cells stained with human anti-MelV serum; Panels C and D are mock and MelV infected cells probed with human anti-KamV serum.

On August 21, during the examination by the local doctor, a throat swab was taken from the index case patient (S1) and sent in viral transport medium (VTM) to the National Public Health Laboratory for virus isolation. After 3 days of culturing, a syncytial cytopathic effect (CPE) was noted in MDCK and Vero cells, but not in Hep-2 cells. After 2 passages in MDCK cells, the virus was able to replicate and cause syncytial CPE in all types of mammalian cell-lines available in the laboratory inclusive of C6/36 (ATCC CRL-1660) cell-line which is of mosquito cell in origin (data not shown). The virus was named Kampar virus (KamV) after the location of the index case. Due to the similar CPE morphology (Figure 1) and cell line susceptibility patterns between KamV and the recently discovered Melaka virus (MelV), which also causes acute respiratory diseases in humans [5], immunofluorescent antibody testing was conducted to examine cross reactivity. As shown in Figure 2, the strong cross reactivity between KamV antigen and human anti-MelV sera, and vice versa, suggested these viruses are closely related. This was later confirmed by further serological characterization and molecular analyses (see below).

### Epidemiological investigation

The small township of Kampar is bordered by forested limestone hills on the eastern side and a scattering of abandoned old tin mining ponds on the other side. S1 is a part-time carpenter and lives with his wife and their youngest son (17 years old) in an old wooden house with metal zinc roof and plain cement floor. His house is situated on reclaimed old tin mining land which is about 80 metres from the central market of Kampar town. Many tall trees, comprising of mango trees, coconut palms and natural wild fruiting trees (in particular, the “sea-almond” or locally named Ketapang trees), are found surrounding his house and between neighbouring houses. A tall fruiting mango tree is planted close to the house which is of less than three feet from the side wall of the house and directly adjacent to the window of the living room. At night, fruit bats were noted to use this mango tree to feed on the “sea-almond” fruits which they obtained from the nearby Ketapang trees. Partially eaten fruits and leftover stones of the fruits abandoned by the fruit bats were noted on the ground just outside the window of the living room or on the roof of the house. Only occasionally, the fruit bats were noted to feed on the ripened mangoes. During night time, S1 and his wife often rested in the living room while watching television. There was no recollection of fruit bats flying into his house or roosting inside the house. There was also no record of dead fruit bats being found in the house compound.

It is not clear at the present time whether KamV is carried by a specific fruit bat species or by multiple bat species circulating in the region. A preliminary survey of bat sera collected in peninsular Malaysia from our previous studies indicated a low prevalence of KamV-specific antibodies in at least two different bat species, *Pteropus vampyrus* (1/55) and *Pteropus hypomelaunus* (2/77). Considering that the closely related PulV virus was isolated from *Pteropus hypomelaunus* [7] and NBV from *Pteropus poliocephalus* [6], it seems likely that these orthoreoviruses may have a broad host range among different bats. It should be noted that smaller fruit bats, such as *Eonycteris spelaean* and *Cynopterus brachyotis*, are more commonly found in the areas where KamV and MelV were discovered. Further field study is required to elucidate the bat species mainly responsible for the spill over of these viruses into human population in Malaysia.

The family keeps a couple of chickens in the backyard and a few pet birds in the patio. A week prior to the onset of S1's illness, his wife bought 6 chickens from a neighbour and one of them died on day 2 of illness.

On follow-up investigation, venous blood samples were taken from S1, his wife (S3) and the doctor (S2) who examined him on August 21. All sera were tested for the presence of antibodies against KamV by immunofluorescent antibody testing using KamV-infected MDCK cells. As indicated by the results in Table 2, anti-KamV antibodies were present in all three subjects, with the index case patient showing the highest level of IgG antibodies.

**Table 2:** Serological response (IgM and IgG) of the index patient and contacts against Kampar virus by indirect immunofluorescent assay*.

Person	Titre of IgM against Kampar virus (dilution)	
	1^st^ serum specimen	2^nd^ serum specimen
S1 (patient)	1∶40	1∶80
S2 (patient's doctor)	ND	1∶20
S3 (patient's wife)	1∶10	ND

	Titre of IgG against Kampar virus (dilution)	
S1 (patient)	1∶80	1∶1280
S2 (patient's doctor)	ND	1∶320
S3 (patient's wife)	1∶80	1∶80

* For S1 and S2, the serum samples were taken on 28 August and 26 September, respectively; for S2, the serum sample was taken on 25 September.

To exclude the possibility that KamV was just a passenger virus which might be commonly present in the human population in this region and was not the cause of the observed sickness in S1 and S2, we have since conducted serological surveillance of 31 human serum samples collected in the same region for an unrelated study in early 2008. None of these sera had any specific IgM antibodies against KamV and only one serum sample contained low level (1∶80) of KamV-reactive IgG antibodies.

### Analysis of cross-neutralization patterns

To further establish the antigenic relationship between KamV and three other known orthoreoviruses in the NBV species group, a four way cross-neutralization was conducted as described in the Materials and Methods with the results presented in Table 3. It is clear that all of the four viruses share significant antigenic relatedness as evident from the cross-neutralization activities of each serum. The failure of any of the four sera to neutralize the control virus, mammalian reovirus 3 (MRV3), and of the MRV3 serum to neutralize any of the four viruses confirmed the specificity of the assay. While the KamV human serum neutralized the homologous virus better than heterologous viruses, such a difference was not observed for the MelV human serum, which seemed to be equally effective in neutralizing all four viruses.

**Table 3:** Cross-neutralization analysis.

Type of serum sample used	Virus used for VNT				
	KamV	MelV	PulV	NBV	MRV3
KamV human S1 (infection)	1∶400	1∶50	1∶100	1∶100	<1∶25
MelV human (infection)	1∶50	1∶50	1∶50	1∶50	<1∶25
PulV rabbit (hyper-immune)	1∶3200	1∶1600	1∶1600	1∶1600	<1∶25
NBV rabbit (hyper-immune)	1∶3200	1∶6400	1∶3200	>1∶6400	<1∶25
MRV3 goose (hyper-immune)	<1∶25	<1∶25	<1∶25	<1∶25	1∶800

### Molecular and phylogenetic characterization

**Figure 3:**
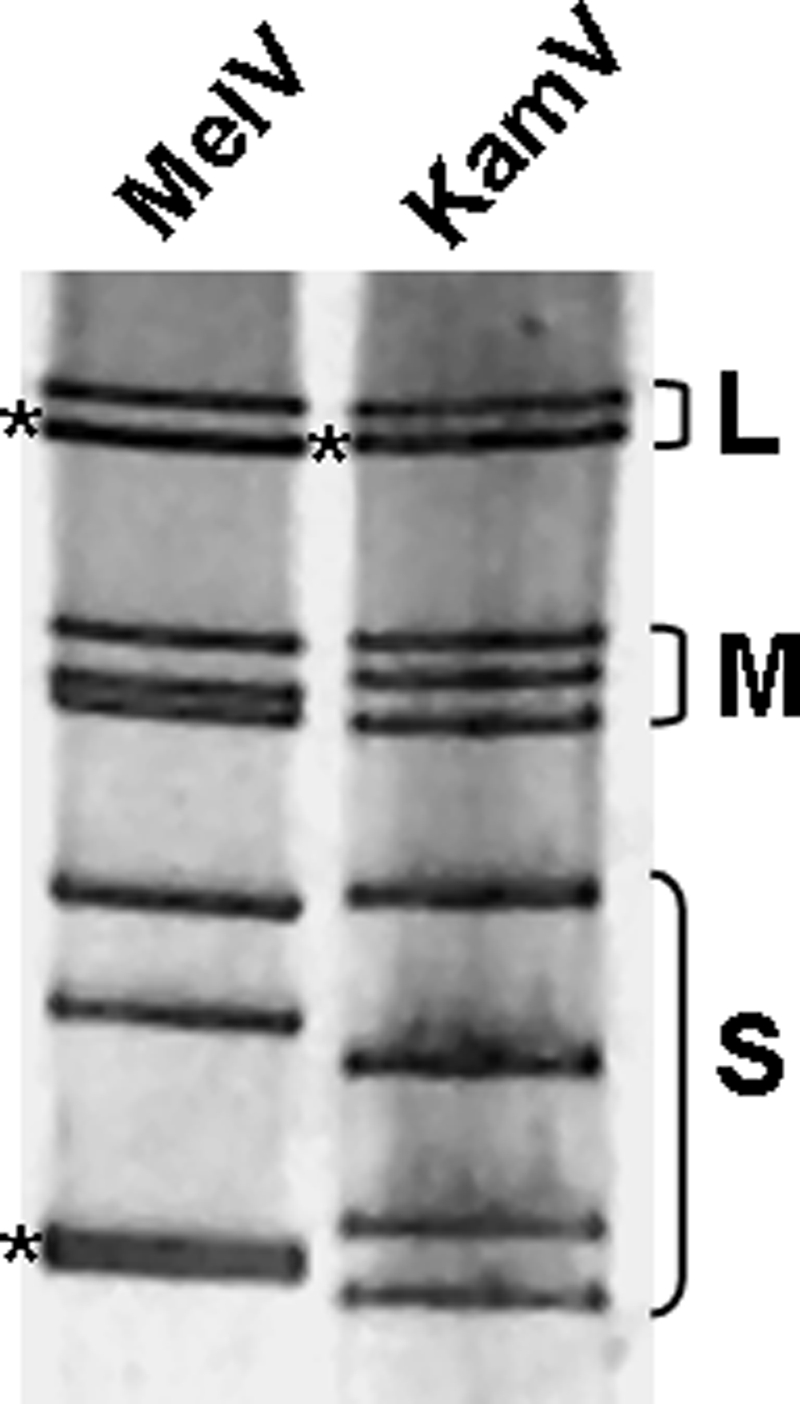
Comparison of genome segment profiles. Genome segments of MelV and KamV were separated on a 10% SDS-polyacrylamide gel. The classes of genome segments (L, M and S) are labeled on the right and the asterisks (*) indicate co-migrating bands where more than one segment is present.

**Figure 4:**
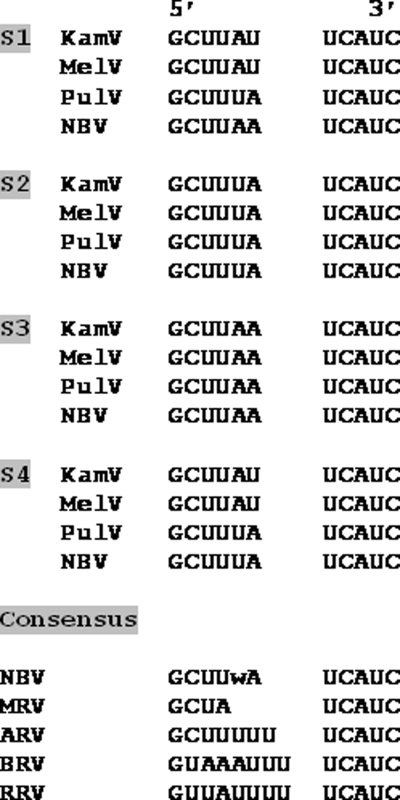
Comparison of genome terminal sequences of different orthoreoviruses. Shown from 5′ end (left) to 3′ end (right) is the sense strand (protein coding strand) of the small RNA genome segments. The consensus sequence for each virus species is shown at the bottom with all viruses in the same genus having the same 3′ terminal sequence.

**Figure 5:**
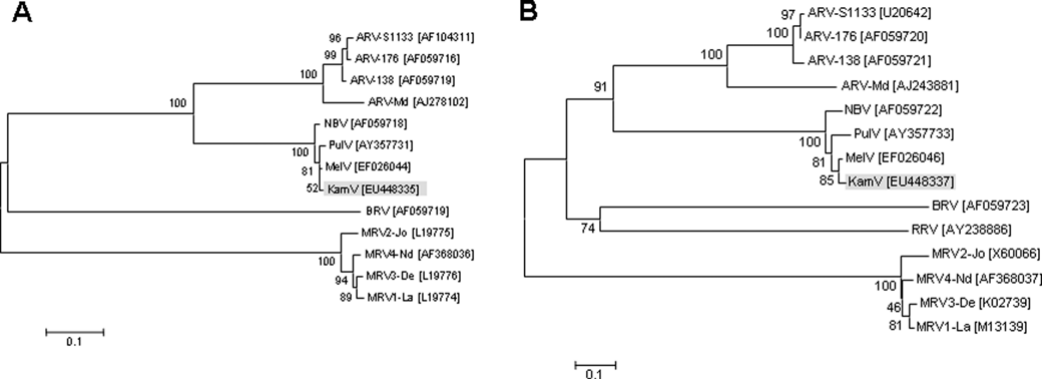
Phylogenetic trees of orthoreoviruses based on deduced amino acid sequence of the major inner capsid or sigma 1 (A) and outer capsid or sigma 2 (B) proteins. GenBank accession number for each sequence is given in brackets next to the abbreviated virus name. Abbreviations: ARV, avian reovirus; BRV, baboon reovirus; KamV, Kampar virus; MRV, mammalian reovirus; MelV, Melaka virus; NBV, Nelson bay virus; PulV, Pulau virus; RRV, reptilian reovirus. Numbers at nodes indicate levels of bootstrap support calculated from 1000 trees.

The close genetic relationship between KamV and MelV was confirmed by the comparison of genome segment mobility or electropherotypes using SDS-PAGE. The two viruses have similar but not identical RNA genome segment profiles (Figure 3). The genetic relatedness of KamV and three other viruses in the NBV species group was further corroborated by the following molecular characteristics: (i) The deduced protein products encoded by the four small (S) genome segments of these viruses are very similar in size and share significant levels of sequence identity (Table 4); (ii) One S-class genome segment of orthoreoviruses may be polycistronic, and its coding arrangement is used as a useful molecular marker for differentiation of different species groups (Duncan et al., 2004). The S1 segment of KamV has an identical coding arrangement to that of the three other viruses (data not shown); (iii) Orthoreoviruses have highly conserved genome terminal sequences at the 5′ end of the positive sense RNA which can be used as a genetic marker for virus classification [3]. For KamV, the consensus sequence is 5′ GCUUww (w = U or A), which is identical to that of MelV, PulV and NBV (Figure 4), but different from other orthoreoviruses. The evolutionary relationship between KamV and other known orthoreoviruses was investigated by conducting phylogenetic analyses for all the proteins encoded by the S-class genome segments. Representative phylogenetic trees based on deduced amino acid sequences of the major outer capsid protein and the major inner capsid protein are shown in Figure 5A and 5B, respectively. It is evident that KamV is a close member of the NBV species. Similar trees were obtained based on other proteins (data not shown).

**Table 4:** Sequence identity and protein size (aa numbers) of deduced KamV protein products in comparison to those from the other three viruses.

KamV Segment/Protein	MelV	PulV	NBV
S1/p10 (95)	93% (95)	94% (95)	72% (95)
S1/p17 (142)	82% (142)	81% (142)	49% (140)
S1/Sigma C (331)	55% (328)	51% (327)	42% (323)
S2/Sigma 1 (416)	99% (416)	98% (416)	97% (416)
S3/NS (367)	98% (367)	99% (367)	96% (367)
S4/Sigma 2 (361)	97% (361)	91% (361)	91% (361)

## Discussion

Respiratory tract infections remain the main infectious disease of humans and account for a large proportion of public health spending worldwide. Despite recent success in discovery of novel respiratory pathogens during the last decade [16]–[18], there is still a substantial proportion of RTIs remaining undiagnosed.

Isolation of MelV from a patient suffering acute respiratory disease marked a new beginning of linking orthoreoviruses to acute human RTIs [5]. This was especially significant in that epidemiology studies indicated that MelV originated from bats. The last decade has experienced a surge in the discovery of emerging viruses of bat origin, several of which have had significant impact on human and animal heath, tourism and trade [8], [19]–[21]. Although the exact reason is not fully known, it seems that bats are an ideal reservoir for a number of zoonotic viruses covering many different virus families.

In this context, the isolation of KamV confirms the observed trend of zoonotic virus emergence out of bat populations in the pacific region. Although there is no direct evidence to prove the origin of KamV, the close genetic relationship of KamV with MelV, PulV and NBV and the location of the house of the index case in close proximity to fruit trees frequently visited by fruit bats indicated a high probability that KamV is also a bat-borne virus. Subsequent serological surveillance confirmed low levels of antibodies to KamV circulating in two bat species commonly found in peninsula Malaysia.

Retrospective serological studies indicated that the index has transmitted the virus to at least one other person, his doctor, presumably via contact droplet nuclei or aerosol. It is more difficult to assess how his wife was infected by KamV although serological results suggested she could have been infected earlier in the same manner as her husband.

The risk of virus spillover from bats as a result of increasing encroachment by animals and humans into bat habitats is enhanced by the wide distribution of a large number of bat species and the seemingly great genetic diversity of newly emergent bat-borne viruses. This is true for henipaviruses [22]–[25], coronaviruses [26], [27], and now orthoreoviruses. Henipaviruses were initially discovered in pteropid bats, but a subsequent seroprevalence survey indicated that henipaviruses or henipa-like viruses are also circulating among non-pteropid bats [28]–[30]. Since the discovery of SARS-like coronaviruses in horseshoe bats, a large number of new coronaviruses have been detected in many different bat species [26], [27], [31]–[35]. It is important to conduct similar serological and virological surveillance studies for bat orthoreoviruses to better understand their geographic distribution, their host range and their genetic diversity. Such information will be essential for an effective risk assessment of future spill over events and potential large scale outbreaks.

**Figure 6:**
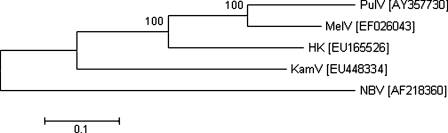
Phylogenetic trees of orthoreoviruses based on partial amino acid sequence of the cell attachment or sigma C protein (aa 1–324). GenBank accession number for each sequence is given in brackets next to the abbreviated virus name. Abbreviations: HK, reovirus strain HK23629/07; KamV, Kampar virus; MelV, Melaka virus; NBV, Nelson bay virus; PulV, Pulau virus. Numbers at nodes indicate levels of bootstrap support calculated from 1000 trees.

During the preparation of this manuscript, the partial S segment sequences of a new orthoreovirus were made available in the GenBank by a group in Hong Kong (GenBank accession numbers EU165526 and EU170365-7). This virus, tentatively named reovirus strain HK23629/07, was isolated from a patient suffering an acute respiratory infection. Phylogenetic analysis of the available sequences confirmed that the HK strain is very closely related to other members of the NBV group. A closer look at the phylogenetic tree based on the most variable protein, cell attachment protein sigma C (Figure 6), indicated that the Malaysian bat isolate (PulV) is more closely related to the three human isolates (MelV, KamV and HK) than to the Australian bat isolate (NBV). This suggests that geographic location, rather than the host of isolation, is a more important determinant for genetic relatedness. Although the exact reservoir species of KamV is yet to be determined, preliminary serological studies suggest that KamV is likely able to infect multiple fruit bat species in Malaysia. The isolation of three related viruses from human patients within such a short period and the close relation between bat and human isolates would suggest that spill over by this group of orthoreoviruses is not an uncommon event and systematic surveillance among RTI patients is needed to provide a more accurate assessment of this newly discovered viral infection among human population in different countries in this region and beyond.

In conclusion, the discovery and characterization of KamV corroborate our previous work on MelV and demonstrate the increasing risk posed by unknown bat viruses which are capable of infecting and causing disease in humans. This further highlights the urgent need to systematically survey bat-borne viruses in the international community so as to enable us to conduct more effective risk assessment, to provide forecast for potential future outbreaks and to devise better prevention and control strategies.

## Materials and Methods

### Virus isolation and characterization

A throat swab was taken from the patient at the time of his first clinical examination and transported in viral transport medium (VTM) to the National Public Health Laboratory for virus isolation. The sample was treated with antibiotics (C. penicillin 100,000 I.U./ml and streptomycin 100 µg/ml) for an hour before being inoculated in duplicate (100 µl and 200 µl, respectively) into freshly confluent monolayers of MDCK (ATCC, CCL-34), Vero (ATCC, CCL-81) and Hep-2 (ATCC, CCL-23) cells cultured in a 24-well tissue culture plate. The plate was incubated at 37°C in 5% CO_2_ and examined daily for the presence of CPE in cultured cells. Supernatant from cultures with visible syncytial cytopathic effect (CPE) after 3 days was taken for further analysis by serial passage in different cell lines available in the laboratory.

The investigation conducted in this study was approved by the ethics committee of the Malaysian National Public Health Laboratory. All patients (subjects) in this manuscript have given written informed consent (as outlined in the PLoS consent form) to publication of their case details. No identification of the subjects is to be revealed in any publication.

### Serological investigation

Immunofluorescence antibody testing (IFAT) and virus neutralization assay were conducted as previously described [5]. Briefly, for IFAT a freshly confluent monolayer of MDCK was infected with KamV and at full CPE, the infected cells were harvested, washed four times and suspended in sterile PBS at a cell concentration of approximately 3000 cells per millilitre. An aliquot of the infected cell suspension was carefully spotted onto each well of Teflon coated slides, followed by air-drying over a warm plate and subsequent fixation in cold acetone for 10 min. Serial 2-fold dilutions of serum samples were then added to detect specific reactivity. For detection of IgM, IgG was removed by absorption with protein A prior to serum dilution. Bound antibodies were detected using fluorescein conjugated rabbit anti-human IgM or IgG (Dako, USA). Specific reactivity/labelling were read under a UV fluorescence microscope (Olympus BX50, Japan). For VNT, serial 2-fold dilutions of control and test sera were prepared in duplicate starting at 1∶10. An equal volume of virus working stock containing 150 TCID_50_ was added to the diluted sera and incubated for 30 min. The pre-incubated virus/serum mix was added to confluent cell monolayers and incubated for 1 h. The inoculum was removed, monolayers washed three times with PBS and cell media replaced. Ability of sera to neutralize virus was determined by scoring the extent of CPE observed in duplicate wells three days later.

### Characterization of genome segments

Virion from culture supernatant was harvested and concentrated by centrifugation and viral RNA was extracted and purified using the QIamp viral RNA kit (Qiagen, Germany). For each virus, an aliquot of 15 µg RNA was run on a 10% SDS-polyacrylamide/Bis gel under denaturing and reducing conditions at 150 V for 4 hrs at room temperature. The gel was washed with distilled water, stained with ethidium bromide before the photo was taken.

### Sequence and phylogenetic analysis

Extraction and purification of dsRNA and synthesis of randomly primed cDNA were carried out as previously described [5], [7], [36]. The majority of sequence information was obtained by PCR with primers designed from conserved regions of MelV, PulV and NBV (primer sequences will be supplied upon request). To obtain the genome segment terminal sequences, a two-step PCR strategy was used. First, the single primer amplification technique or SPAT [36] was used to generate primary PCR product followed by a semi-nested PCR using the combination of KamV genome segment-specific primers and the adaptor-specific primer used in SPAT. PCR products were sequenced directly without cloning. All regions of the genome segments were sequenced at least three times. Phylogenetic analysis was conducted using the MEGA4 software package [37]. Phylogenetic trees were constructed using the neighbour-joining algorithm with bootstrap values determined by 1,000 replicates. Complete genome sequences of the four S segments were deposited in GenBank under accession numbers EU448334 to EU4488337 for S1, S2, S3 and S4 segments, respectively.
